# Calcium pyrophosphate deposition disease: A case report with bilateral involvement of the temporomandibular joints and concurrence of psoriatic arthritis

**DOI:** 10.1002/ccr3.2715

**Published:** 2020-02-05

**Authors:** Lado Lako Loro, Tore Bjørnland

**Affiliations:** ^1^ Section of Maxillofacial Surgery Department of Ophthalmology, Otolaryngology and Maxillofacial Surgery Møre and Romsdal Hospital Trust Ålesund Hospital Ålesund Norway; ^2^ Department of Oral Surgery and Oral Medicine Faculty of Dentistry University of Oslo Oslo Norway

**Keywords:** calcium pyrophosphate dehydrate deposition disease, psoriatic arthritis, temporomandibular joint

## Abstract

Calcium pyrophosphate dehydrate deposition (CPDD) disease very rarely affects the temporomandibular joint (TMJ). It may resemble synovial chondromatosis, chondrosarcoma, chondroblastoma, or a parotid tumor. Clinical examination, CT, and MRI are important in making the correct diagnosis. Surgical removal of CPDD is necessary with or without excision of the TMJ.

## Graphical abstract

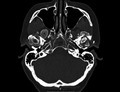

## 1 INTRODUCTION

Calcium pyrophosphate dehydrate deposition (CPDD) disease is a rare disease in the temporomandibular joint (TMJ). It usually affects other joints, and patients are usually over the age of 60.^1^, ^2^ Only few cases with CPDD in the TMJ have been reported.^3^, ^4^, ^5^

The etiology of CPDD still remains unclear. However, it has been reported that advanced age, rheumatoid arthritis,^6^ osteoarthritis,^2^ long‐standing gout,^7^ and surgery 8, 9 may be risk factors for CPDD. A higher incidence of CPDD in patients taking diuretics may indicate a relationship with diuretic‐induced hypomagnesaemia.^10^

The clinical picture of CPDD in the TMJ may be unspecific, patients may present with reduced mandibular motion, swelling, and pain in the TMJ and surrounding structures.^3^, ^4^, ^5^ We report a case with bilateral TMJ involvement in a woman with psoriatic arthritis with clinical, radiographic, and intraoperative findings.

## 2 CASE HISTORY

**Figure 1:**
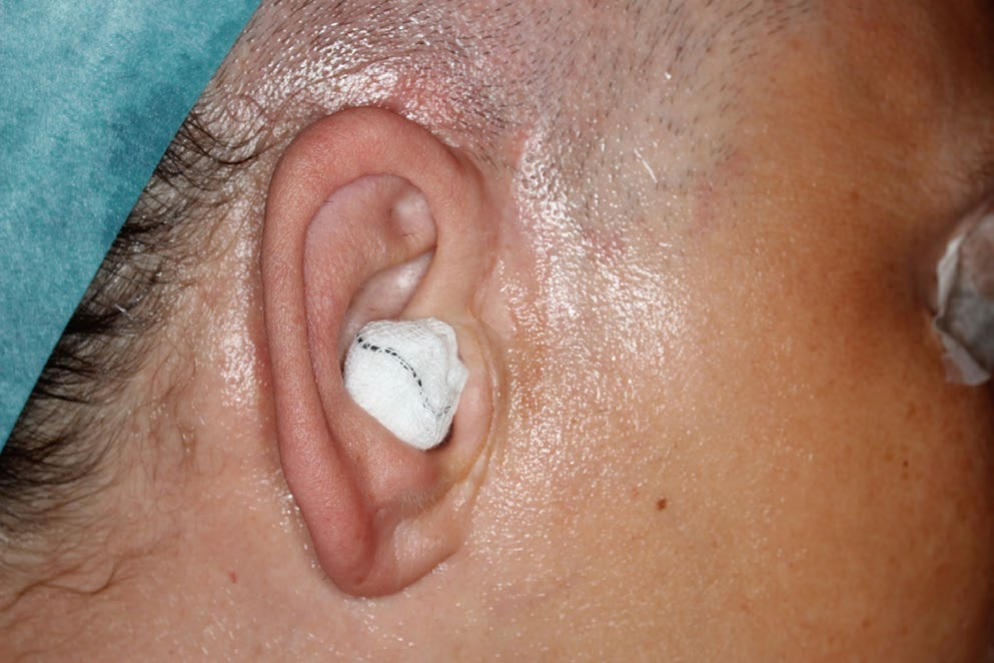
Preoperative photograph showing diffuse preauricular swelling on the right side

A 40‐year‐old woman with a medical history of asthma, hypertension, and psoriatic arthritis with involvement of both TMJs diagnosed over 20 years ago. She was on the following medications: selective immune suppressor (Araba), Angiotensin II receptor blocker (Diovan), leukotriene receptor antagonist (Singulair), antihistamines (Arius tablets and Livostin eye drops), corticosteroid (Avamys nasal spray), estrogen replacement (Progynova), proton pump inhibitor (Pantoprazole), sedative (Zolpidem), and analgesics (paracetamol, codeine/paracetamol and oxycodone). The patient presented with a swelling in the left preauricular region. She had a long past history of TMJ pain and limitation of jaw movements and had, 14 years earlier, undergone bilateral discectomy and synovectomy. Three years previously an arthroplasty and synovectomy in the right TMJ was done because of severe pain and significantly reduced TMJ function. A diffuse swelling over the right preauricular region was observed (Figure 1) and a year later the same surgery was performed on the left TMJ with interpositional dermis‐fat graft placement. During the operation on the left side, a whitish/yellowish chalky material was observed and removed from the joint. A sample was taken and sent for histopathological evaluation. The biopsy report showed massive dystrophic calcium deposits surrounded by a palisading histiocytic reaction with epithelioid and multinuclear giant cells.

**Figure 2:**
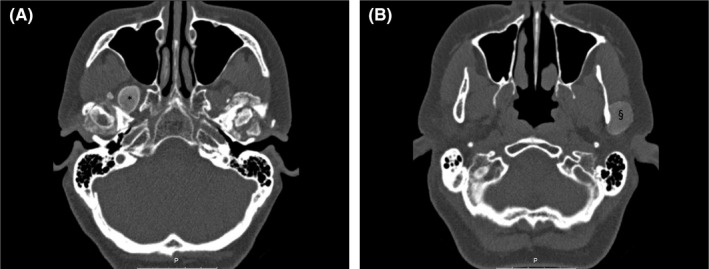
A, Axial CT scan showing extensive calcification with deformation and arthrosis in both temporomandibular joints and surrounding structures including a well‐circumscribed calcified nodule in the right pterygopalatine fossa region (*). B, Axial CT scan showing well‐defined calcified nodule (§) in the left preauricular region caudal to the temporomandibular joint

**Figure 3:**
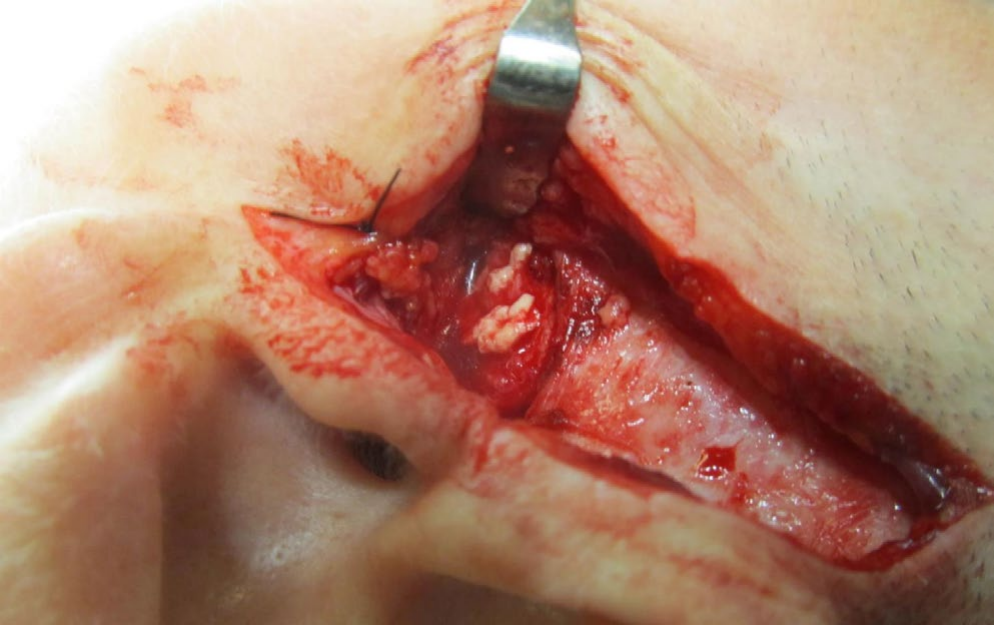
Intraoperative photograph showing calcium deposits over the left temporomandibular joint

The patient's range of jaw movements improved after surgery but she still complained of severe TMJ pains. Four months after surgery, she presented with a preauricular swelling about 1.5 × 2.0 cm on the left side. The nodule was hard, well defined, and tender on palpation. The overlying skin was normal. A parotid tumor was suspected; MRI was ordered, and fine needle aspiration cytology (FNAC) was undertaken. The MRI examination showed a well‐defined encapsulated lesion related to the TMJ, possibly an inflammatory pseudo‐tumor, parotid tumor, synovial cyst, or postoperative changes. There were no findings typical of parotid tumors, and the FNAC was nonspecific. A new MRI dedicated to the TMJ was taken, which showed progressive bilateral calcification around the TMJ. A diagnosis of calcium pyrophosphate dehydrate deposition disease was suggested. CT scans showed extensive progressive calcification in both TMJs with deformation and arthrosis (Figure 2A). CT findings on the nodule on the left side were most consistent with a pseudo‐tumor with calcification (Figure 2B). The differential diagnosis included synovial chondromatosis, chondrosarcoma, and chondroblastoma. The patient was referred for a new serological evaluation of rheumatologic or metabolic disease. The evaluation revealed normal blood values, showing that the CPDD was localized to the jaws only. The tumor on the left side and part of the calcium pyrophosphate deposits (Figure 3) were removed under general anesthesia. The patient continued to have severe pain and progressive limitation of mouth opening and was later operated on and bilateral total TMJ prostheses were placed.

## 3 DISCUSSION

Calcium pyrophosphate dehydrate deposition may occur as a generalized disease or localized to a specific joint. Localized CPDD is believed to occur secondary to trauma or following surgical procedures.^8^, ^9^, ^11^ Our case had CPDD localized to the TMJs after a long history with psoriatic arthritis and multiple TMJ surgeries. Thus, one may speculate that CPDD in our case occurred because of the repeated TMJ operations. There were no clinical or laboratory findings that would indicate an association with metabolic or connective tissue disease. The preauricular tumor presenting on the left side clinically mimicked a parotid tumor but this hypothesis was eliminated after MRI imaging. CPDD tumors may clinically and radiographically resemble synovial chondromatosis or neoplasms such as osteochondroma, chondrosarcoma, and chondroblastoma. A biopsy of the lesion is necessary to rule out or confirm a diagnosis of neoplasm. Synovial chondromatosis and neoplasia could be excluded intraoperatively as there were no characteristic multiple metaplastic nodules of cartilage in the joint. The noncohesive chalky material around the TMJ was in contrast to the hard cohesive masses typical of neoplastic TMJ lesions.^12^

Treatment of CPDD varies according to the presentation and clinical findings. Calcium pyrophosphate crystals cannot be dissolved; therefore, management of crystal‐derived inflammation is important.^13^ Mild cases can be managed with nonsteroidal anti‐inflammatory drugs, colchicine, or glucocorticoids.^13^ Surgical excision is the treatment of choice for CPDD tumor or tophaceous pseudogout cases.^14^, ^15^ Usually, symptoms are relieved and function restored after surgical excision.^12^, ^16^ However, in our case the condition progressed rapidly after surgery with massive bilateral bony erosion around the temporomandibular joint. The choice of treatment in such cases may be total TMJ prosthesis placement to avoid ankylosis. Immediate replacement of the TMJ with total joint arthroplasty following surgical resection of a tumoral CPDD has previously been described.^17^

In conclusion, we report a case of CPDD of the TMJ in a patient with psoriatic arthritis with tophaceous pseudogout presentation, deformation, and bilateral arthrosis. Tumoral CPDD can be confused clinically and radiographically with TMJ neoplasms, biopsies are necessary for the diagnosis. Treatment is by surgical excision in tumor cases and cases with erosion of the TMJ. Total TMJ prosthesis may be necessary to restore function and avoid ankylosis.

## CONFLICT OF INTEREST

None declared.

## AUTHOR CONTRIBUTION

Both authors have contributed equally during the treatment of the patient and in the process of writing of the manuscript.
